# Nomenclatural checklist for *Acromegalomma* species (Annelida, Sabellidae), a *nomen novum* replacement for the junior homonym *Megalomma* Johansson, 1926

**DOI:** 10.3897/zookeys.677.12030

**Published:** 2017-05-29

**Authors:** João Gil, Eijiroh Nishi

**Affiliations:** 1 CEAB-CSIC, Carrer d’accés a la Cala Sant Francesc, 14, 17300 Blanes (Girona), Spain / CCMAR, Universidade do Algarve, Campus Gambelas, 8005-139 Faro, Portugal; 2 College of Education and Human Science, Yokohama National University, Hodogaya Yokohama, 240-8501, Japan

**Keywords:** Fan worms, polychaete, homonymy, new substitute name, nomenclature, taxonomy

## Abstract

*Acromegalomma*, *nomen novum*, is introduced as a replacement name for the polychaete genus *Megalomma* Johansson, 1926 (Annelida, Sabellidae), preoccupied by *Megalomma* Westwood, 1842 (Insecta, Coleoptera, Carabidae). The historical background of the homonymy and a full list with 36 new combinations in the new genus are included, while two species are considered as *species inquirenda*.

## Introduction

The genus *Megalomma* (Annelida, Sabellidae) was established by Johansson (1926) for the usage of *Branchiomma* *sensu* Claparède (1869), based on the species *Branchiomma koellikeri* Claparède, 1869. However, the name *Megalomma* is preoccupied by *Megalomma* Westwood, 1842 (Insecta, Coleoptera), a well-established genus of tiger beetles from the Mascarene Islands. *Megalomma* Johansson, 1926 has no known available or potentially valid synonyms, for which reason, and in accordance with Article 60.3 of the ICZN (1999), it must be replaced by a new substitute name.

## Historical background

The name *Megalomma* was first used as a subgenus of *Cicindela* Linnaeus, 1758 (Insecta, Coleoptera, Carabidae) by Westwood (1842: 203), for the Mauritian species Cicindela (Megalomma) vigilans Westwood, 1842, and raised to the generic level the following year by Lacordaire (1843: 113). The genus is well established and in current use (see Moravec 2007), comprising five species from the Mascarene Islands (West Indian Ocean): *M. fulgens* W. Horn, 1892; *M. janaki* Moravec, 2007; *M. oculatum* (Fabricius, 1799); *M. pierreorum* Deuve, 2000; and *M. viridulum* (Quensel *in* Schönherr, 1806), which includes as a synonym the type species of the genus, *M. vigilans* (Westwood, 1842).

A second genus *Megalomma* was created by Smith (1873: 405) in Hymenoptera (Insecta), to include three new species from Brazil: *Megalomma politum* Smith, 1873, *M. elegans* Smith, 1873, and *M. nigriceps* Smith, 1873. Later Schulz (1906: 200) pointed that it was a junior homonym of *Megalomma* Westwood, 1842 and replaced it by the new name *Megistommum* Schulz, 1906.

In polychaetes, the name *Megalomma* was first used by Johansson (1926: 10), as a replacement name for *Branchiomma* *sensu* Claparède (1869), based on a misinterpretation of Claparède’s work. While discussing the validity of the name *Dasychone* introduced by Sars (1862) for sabellids with eyes on their radioles, Claparède (1869) stated that Kölliker (1858) had already used the name *Branchiomma* for the same group, to include *Amphitrite bombyx* Dalyell, 1853 (renamed as *Branchiomma dalyellii* Kölliker, 1858). However, instead of synonymizing the junior *Dasychone* into *Branchiomma*, Claparède (1869: 162) tried to retain both by redefining the genus *Branchiomma*, with the following justification:

*Quoi qu’il en soit, le nom de* Dasychone *a pris place dans la science, et celui de* Branchiomma *est à peu près oublié. Je pense pourtant pouvoir ressusciter celui-ci, en tenant compte des scrupules de M. Sars, et sans proscrire le nom de* Dasychone. *Dans son mémoire sur le genre* Branchiomma, *M. Kölliker décrit en outre de la* Dasychone Bombyx *une autre espèce qu’il n’a étudiée que d’une manière très-cursive, il est vrai, dans le golfe de Naples, et qui est caractérisée par des yeux à l’extrémité des branchies. Cette espèce que j’ai retrouvée n’est point une* Dasychone. *Elle pourra rester dorénavant l’espèce-type du genre* Branchiomma.

Hence, Claparède (1869) attempted to transfer *Amphitrite bombyx* Dalyell, 1853 to *Dasychone* (see Claparède 1869: 168), while making reference to a short comment by Kölliker (1858: 536) where he recorded that he had observed, but not named nor described in detail, an additional sabellid from Naples with eight compound eyes near the tips of the radioles:

*Schon im Jahre 1842 kam mir in Neapel ein kleiner Kopfkiemer unter die Augen, der an seinen Kiemen 8 zusammengesetzte Sehorgane trug. Leider war es mir damals, da ich gerade mit der Verfolgung der Entwicklung der Cephalopoden beschäftigt war, nicht möglich, diese interessante Annelide, von der ich ohnehin nur Ein Individuum erhalten hatte, näher zu verfolgen, und unterliess ich es daher, etwas über dieselbe zu veröffentlichen*.

Consequently, Claparède described this species as *Branchiomma koellikeri* [original spelling *köllikeri* corrected here to *koellikeri* according to Article 32.5.2.1 of the ICZN (1999)], based on the specimens collected by him at the Gulf of Naples, citing (Claparède 1869: 164):

*Je doute à peine que cette espèce soit la même que M. Kölliker a eue sous les yeux. Elle n’est en effet point rare dans le golfe de Naples. M. Kölliker n’indique, il est vrai, que huit filaments branchiaux, tandis que j’en ai compté jusqu’à trente-deux. Mais cela peut ne tenir qu’à une différence d’âge*.

This leaves little doubt that Claparède considered his new species *Branchiomma koellikeri* to be the same species observed previously by Kölliker from Naples and, moreover, that he proposed *B. koellikeri* as the type of his emendation of Kölliker’s genus with the sentence (Claparède 1869: 162): “*Elle pourra rester dorénavant l’espèce-type du genre* Branchiomma.”

Apparently Claparède’s intention was simply to redefine the genus *Branchiomma* to restrict it to the unnamed Kölliker species (= *B. koellikeri* Claparède, 1869, the intended new type species of the redefined genus), as can be inferred by the fact that he wrote “*Genre* Branchiomma *Koellkr. char. em.*” (Claparède 1869: 162).

However, Johansson (1926: 10) considered erroneously that Claparède was formally establishing a new genus, an interpretation that was followed by other authors (e.g. Hartman 1959, Day 1967, Fauchald 1977).

In this way, Johansson (1926: 10) argued that the generic name *Branchiomma* *sensu* Claparède (1869), used for *Branchiomma koellikeri*, could not be accepted, as it was already preoccupied by Kölliker (1858) for the species *Amphitrite bombyx* Dalyell, 1853. A new replacement name was thus necessary, and Johansson introduced for the third time in Zoology the name *Megalomma*, emphasizing the large compound eyes of the genus (Johansson 1926: 10):

*Als Claparède 1869 für seine Art* Köllikeri *die Gattung* Branchiomma *bildete, war der Name schon präokkupiert. Die Gattung* Branchiomma *Claparède muss also einen neuen Namen erhalten. Ich schlage* Megalomma *vor, welcher Name wie* Branchiomma *auf die grossen zusammengesetzen Augen dieser Gattung hindedeutet, doch ohne ihren Charakter als Branchialaugen hervorzuheben* [...].

However, and as stated above, the name *Megalomma* Johansson, 1926 is itself preoccupied by *Megalomma* Westwood, 1842, and a replacement name is necessary. The name *Acromegalomma*, *nomen novum* is here proposed to accomplish this need.

### *Chronology of the genus-level name* Megalomma

1842. Westwood (p. 203): Cincidella (Megalomma) new subgenus (Insecta, Coleoptera, Carabidae), for Cicindella (Megalomma) vigilans Westwood, 1842.

1843. Lacordaire (p. 113): *Megalomma* raised to generic level.

1858. Kölliker (p. 537): *Branchiomma* new genus (Annelida, Sabellidae), for *Amphitrite bombyx* Dalyell, 1853 (renamed as *Branchiomma dalyellii* Kölliker, 1858).

1869. Claparède (p. 162–163): *Branchiomma* redefined (Annelida, Sabellidae), for *Branchiomma koellikeri* Claparède, 1869. *Amphitrite bombyx* Dalyell, 1853 assigned to *Dasychone* Sars, 1862.

1873. Smith (p. 405): *Megalomma* new genus (Insecta, Hymenoptera, Crabronidae), for *Megalomma politum* Smith, 1873, *M. elegans* Smith, 1873, and *M. nigriceps* Smith, 1873.

1906. Schulz (p. 200): *Megistommum* new name (Insecta, Hymenoptera, Crabronidae), replacement name for *Megalomma* Smith, 1873.

1926. Johansson (p. 10): *Megalomma* new genus (Annelida, Sabellidae), to include *Branchiomma* *sensu* Claparède, 1869 (not *Branchiomma* Kölliker, 1858).

Present study. *Acromegalomma* new name (Annelida, Sabellidae), replacement name for *Megalomma* Johansson, 1926.

## Material and methods

To establish the list of new combinations in *Acromegalomma* new name a list of valid *Megalomma* species was compiled based on WoRMS (Bellan 2008), and updated with Tovar-Hernández and Carrera-Parra (2011), Mikac et al. (2013), Capa and Murray (2015) and Giangrande et al. (2015). Synonymies were obtained from Tovar-Hernández and Carrera-Parra (2011). The type of synonymy and its author are provided inside square brackets, following the synonym.

Each new combination is accompanied by the reference of the original description, synonymies, type locality of the species and remarks, when necessary. Type locality is based on the original description, except where indicated. Geolocations of type localities are derived from the original descriptions, being considered an “original geolocation” when the authors provided the coordinates, or “estimated geolocation”, when estimated using Google Earth (www.google.com/earth) from the general geographic limits described by the authors. All geolocations were converted to decimal degrees.

The whereabouts of type material of the new combinations were summarised by Tovar-Hernández and Carrera-Parra (2011) and following publications describing new species (Mikac et al. 2013, Capa and Murray 2015, Giangrande et al. 2015).

While the gender of *Megalomma* and *Acromegalomma* new name is neuter, some names in *Megalomma* had incorrect endings and needed to be emended. Following Article 31.2 of the ICZN (1999), the names of the new combinations and the *species inquirenda* were herein revised to assure they agreed in gender with the generic name with which they are combined. Original names with incorrect endings are indicated with the remark “[*sic*]” following the specific epithet. Endings corrected herein are: *carunculatum* for *carunculata*, *inflatum* for *inflata*, *interruptum* for *interrupta*, *jubatum* for *jubata*, and *longoventrale* for *longoventralis*.

## Systematics

### Order Sabellida Latreille, 1825

#### Family Sabellidae Latreille, 1825

##### Genus Acromegalomma

Taxon classificationAnimaliaSabellidaSabellidae

, nomen novum

Branchiomma [not Kölliker, 1858] — Claparède 1869: 162–163.
Megalomma [junior homonym, not Westwood, 1842] — Johansson 1926: 9–10; Johansson 1927: 130; Perkins 1984: 351–352; Fitzhugh 1989: 76; Knight-Jones 1997: 314; Fitzhugh 2003: 107; Tovar-Hernández and Salazar-Vallejo 2008: 1953–1954; Giangrande and Licciano 2008: 208; Capa and Murray 2009: 204–205; Tovar-Hernández and Carrera-Parra 2011: 14–15; Mikac et al. 2013: 1514; Capa et al. 2014: 27–28; Giangrande et al. 2015: 522–523.

###### Type species.

The type species of the new genus is *Branchiomma koellikeri* Claparède, 1869 (junior synonym of *Sabella lanigera* Grube, 1846), according to recommendation 60A of the ICZN (1999). Type by monotypy, established by Johansson (1926).

###### Type material.

Following his principle of basing observations and descriptions only on live organisms Édouard Claparède did not designate type material or deposit specimens in museums or collections (Fauchald 1989). However, Knight-Jones (1997) refers the existence of a type of *Branchiomma koellikeri* Claparède, 1869 deposited at the Zoological Museum of Berlin (currently the Museum für Naturkunde Berlin), with the reference number ZMB 6387. Tovar-Hernández and Carrera-Parra (2011) refer this specimen as being a syntype. Although the designation of a lectotype for *B. koellikeri* is desirable, it is out of the scope of the present work.

###### Etymology.

The name of the new genus is composed by combining the Greek terms for *acro*, meaning “tip end” or “extremity of a body”, *mega*, meaning “big” or “large”, and the suffix –*omma*, a noun meaning “eye”, and referring to the big compound eyes located on the radiolar subdistal region, typical of the genus.

###### Gender.

Neuter.

###### Remarks.

The publication date of the genus *Megalomma* Johansson should be considered as “1926”. It was generally accepted as being “1927” until Tovar-Hernández and Salazar-Vallejo (2008) pointed out that the name had been introduced in a previous publication by the same author, referring the date as “1925”. In fact, the last page of this publication states “*Tryckt den 5 november 1925*” (“Printed the 5 November 1925”) and in the following line “*Uppsala 1925. Almquist & Wiksells Boktryckeri-A.-B.*” This date is also present in existing reprints of the paper. However, the bounded volume comprising the article provides the publication date as “*Häfte 2 inneh. A N:o 6-12*, [...] *utkom den 5 juni 1926*” (“Booklet 2 cont. A No. 6-12, [...] published 5 June 1926”). This booklet includes article 7A by Johansson, where the name *Megalomma* is introduced for polychaetes. Hence, the work was printed in 5 November 1925, but published only in the following year, on 5 June 1926.

The genus *Acromegalomma*, *nomen novum* is represented by 36 valid species, all of them new combinations.

##### List of new combinations in the genus *Acromegalomma* new name

###### Acromegalomma acrophthalmos

Taxon classificationAnimaliaSabellidaSabellidae

(Grube, 1878) comb. n.

Sabella acrophthalmos Grube 1878: vii, 258–259.

####### Type locality.

Singapore (1.25°, 103.85°; estimated geolocation) or Philippines (12.22°, 121.77°; estimated geolocation).

####### Remarks.

The type locality of the species was first stated as being “probably Singapore” (“*wahrscheinlich von Singapore*”; Grube 1878: vii), and later in the same publication, as “Philippines” (“*Von den Philippinen*”; Grube 1878: 258).

###### Acromegalomma adriaticum

Taxon classificationAnimaliaSabellidaSabellidae

(Giangrande, Caruso, Mikac & Licciano, 2015) comb. nov.

Megalomma adriaticum Giangrande et al. 2015: 526–528, figs 6–8.

####### Type locality.

Brindisi, Italy, South Adriatic Sea (40.65°, 17.95°; original geolocation).

###### Acromegalomma bioculatum

Taxon classificationAnimaliaSabellidaSabellidae

(Ehlers, 1887) comb. n.

Branchiomma bioculatum Ehlers 1887: 260–263, plate 53 figs 1–9.

####### Type locality.

West of Dry Tortugas, Straits of Florida (24.6181°, -83.0517°; original geolocation).

###### Acromegalomma carunculatum

Taxon classificationAnimaliaSabellidaSabellidae

(Tovar-Hernández & Salazar-Vallejo, 2008) comb. n.

Megalomma carunculata [*sic*] Tovar-Hernández and Salazar-Vallejo 2008: 1957–1961, figs 1–2.

####### Type locality.

Punta Manzanillo, Acapulco, Guerrero, Mexican Pacific (16.842°, -99.910°; estimated geolocation).

###### Acromegalomma cinctum

Taxon classificationAnimaliaSabellidaSabellidae

(Fitzhugh, 2003) comb. n.

Megalomma cinctum Fitzhugh 2003: 108–116, figs 1–10, 14C.

####### Type locality.

Hungtou Yu (Orchid Island), northern coastline, about 1 km east of Langtao Village, Taiwan, Pacific Ocean (22.0794°, 121.5369°; original geolocation).

###### Acromegalomma circumspectum

Taxon classificationAnimaliaSabellidaSabellidae

(Moore, 1923) comb. n.

Branchiomma circumspectum Moore 1923: 239–241, plate XVIII figs 41–42.

####### Type locality.

Between S. 35° W, 3.5 miles (5.6 km) and S. 43° W, 5.2 miles (8.4 km) off Brockway Point, Santa Rosa Island, Channel Islands, California, Pacific coast of the USA (34.02°, -120.22°; estimated geolocation).

###### Acromegalomma claparedei

Taxon classificationAnimaliaSabellidaSabellidae

(Gravier, 1906) comb. n.

Branchiomma claparedei Gravier 1906: 39–40.

####### Type locality.

Syntypes collected at the reef of Marabout (11.611°, 43.132°; estimated geolocation), at Djibouti Bay, and the “Grand Récif” (11.736°, 43.235°; estimated geolocation), Moucha Islands, both at the Gulf of Tadjoura, Gulf of Aden, Indian Ocean.

####### Remarks.

Gravier introduced the name *Branchiomma claparedei* as new twice, first in 1906 (Gravier 1906: 39) and again in 1908 (Gravier 1908b: 91). This caused some confusion, inducing some authors in error, by considering the correct publication date as being 1908, while overlooking the smaller 1906 publication (e.g. Tovar-Hernández and Carrera-Parra 2011). The correct publication date is therefore “1906” (see also Wehe and Fiege 2002).

###### Acromegalomma coloratum

Taxon classificationAnimaliaSabellidaSabellidae

(Chamberlin, 1919) comb. n.

Potamilla colorata Chamberlin 1919b: 21.

####### Type locality.

Laguna Beach, California, Pacific coast of the USA (33.542°, -117.786°; estimated geolocation).

####### Synonym.

*Potamilla clara* Chamberlin 1919b: 20 [subjective synonymy by Tovar-Hernández and Carrera-Parra (2011)].

###### Acromegalomma fauchaldi

Taxon classificationAnimaliaSabellidaSabellidae

(Giangrande, Licciano & Gambi, 2007) comb. n.

 Giangrande et al. 2007: 46–47, fig. 2.

####### Type locality.

Lagoon side of Carrie Bow Cay, Belize, Caribbean Sea (16.803°, -88.085°; estimated geolocation).

###### Acromegalomma georgiense

Taxon classificationAnimaliaSabellidaSabellidae

(Tovar-Hernández & Carrera-Parra, 2011) comb. n.

Megalomma georgiense Tovar-Hernández and Carrera-Parra 2011: 56–58, fig. 26A–L.

####### Type locality.

Off Georgia, Atlantic coast of the USA (30.95°, -79.9667°; original geolocation).

###### Acromegalomma gesae

Taxon classificationAnimaliaSabellidaSabellidae

(Knight-Jones, 1997) comb. n.

Megalomma gesae Knight-Jones 1997: 318–319, fig. 3.

####### Type locality.

La Herradura, Estero Jaltepeque, El Salvador, Pacific Ocean (13.303°, -88.902°; estimated geolocation).

####### Synonym.

*Potamilla bioculata* Hartmann-Schröder 1959: 175–176, figs 183–188 [objective synonymy by Knight-Jones (1997); *Megalomma gesae* is a new name for *P. bioculata*].

###### Acromegalomma heterops

Taxon classificationAnimaliaSabellidaSabellidae

(Perkins, 1984) comb. n.

Megalomma heterops Perkins 1984: 359–363, figs 42–43.

####### Type locality.

Hutchinson Island, Florida, Atlantic Ocean (27.345°, -80.2133°; original geolocation).

###### Acromegalomma inflatum

Taxon classificationAnimaliaSabellidaSabellidae

(Capa & Murray, 2009) comb. n.

Megalomma inflata [*sic*] Capa and Murray 2009: 217–218, figs 4G–H, 5D, 11.

####### Type locality.

Southeast of Bate Bay, New South Wales, Australia (-34.0667°, 151.2167°; original geolocation).

###### Acromegalomma interruptum

Taxon classificationAnimaliaSabellidaSabellidae

(Capa & Murray, 2009) comb. n.

Megalomma interrupta [*sic*] Capa and Murray 2009: 210–212, figs 2J–M, 4E–F, 5B, 7, 8.

####### Type locality.

One Tree Island, Queensland, Australia (-23.5°, 152.0833°; original geolocation).

###### Acromegalomma jubatum

Taxon classificationAnimaliaSabellidaSabellidae

(Capa & Murray, 2015) comb. n.

Megalomma jubata [*sic*] Capa and Murray 2015: 128–130, fig. 12.

####### Type locality.

MacGillivray Reef, Lizard Island, Queensland, Australia (-14.6569°, 145.4947°; original geolocation).

###### Acromegalomma kaikourense

Taxon classificationAnimaliaSabellidaSabellidae

(Knight-Jones, 1997) comb. n.

Megalomma kaikourense Knight-Jones 1997: 320–321, fig. 5.

####### Type locality.

Point Kean near Kaikoura, east coast of South Island, New Zealand (-42.425°, 173.715°; estimated geolocation).

###### Acromegalomma lanigerum

Taxon classificationAnimaliaSabellidaSabellidae

(Grube, 1846) comb. n.

Sabella lanigera Grube 1846: 51–53, plate II fig. 1.

####### Type locality.

Unknown.

####### Synonyms.

*Branchiomma köllikeri* Claparède 1869: 163–164, plate XXII fig. 4 [subjective synonymy by Knight-Jones (1997)]. *Branchiomma vesiculosum neapolitana* Claparède 1869: 164–166, plate XXII fig. 5 [subjective synonymy by Giangrande and Licciano (2008)].

####### Remarks.

The species was described based on a single specimen (T-ZMB 136) deposited at the Zoological Museum of Berlin (currently the Museum für Naturkunde Berlin), from an unknown location (Grube 1846: 51).

###### Acromegalomma lobiferum

Taxon classificationAnimaliaSabellidaSabellidae

(Ehlers, 1887) comb. n.

Branchiomma lobiferum Ehlers 1887: 254–259, plate 53, figs 10–15 (figure 15 numbered as 16 in plate 53).

####### Type locality.

Key West, Florida, Gulf of Mexico (24.54°, -81.80°; estimated geolocation).

###### Acromegalomma longoventrale

Taxon classificationAnimaliaSabellidaSabellidae

(Giangrande, Caruso, Mikac & Licciano, 2015) comb. n.

Megalomma longoventralis [*sic*] Giangrande et al. 2015: 524–526, figs 3–5.

####### Type locality.

Rovinj, coastal station near the Island Banjole, Croatia, North Adriatic Sea (45.095250°, 13.619283°; original geolocation).

###### Acromegalomma messapicum

Taxon classificationAnimaliaSabellidaSabellidae

(Giangrande & Licciano, 2008) comb. n.

Megalomma messapicum Giangrande and Licciano 2008: 213–214, figs 5G–H, 6.

####### Type locality.

Brindisi, Italy, Adriatic Sea (40.65°, 17.96°; estimated geolocation).

###### Acromegalomma miyukiae

Taxon classificationAnimaliaSabellidaSabellidae

(Nishi, 1998) comb. n.

Megalomma miyukiae Nishi 1998: 53–54, figs 1–4.

####### Type locality.

Ao Tang Khen, Phuket, Thailand, Andaman Sea (7.8185°, 98.4144°; estimated geolocation).

###### Acromegalomma modestum

Taxon classificationAnimaliaSabellidaSabellidae

(Quatrefages, 1866) comb. n.

Sabella modesta Quatrefages 1866: 451–452.

####### Type locality.

Lima, Peru, Pacific Ocean (-12.07°, -77.15°; estimated geolocation).

####### Synonym.

*Potamilla anophthalma* Hartmann-Schröder 1960: 41–43, figs 89–91 [subjective synonymy by Knight-Jones (1997)].

###### Acromegalomma multioculatum

Taxon classificationAnimaliaSabellidaSabellidae

(Fitzhugh, 2002) comb. n.

Megalomma multioculatum Fitzhugh 2002: 401–405, figs 34A–C, 35A–B, 36A–D, 37.

####### Type locality.

Thailand, Andaman Sea (08.5°, 98.1°; original geolocation).

###### Acromegalomma mushaense

Taxon classificationAnimaliaSabellidaSabellidae

(Gravier, 1906) comb. n.

Branchiomma mushaensis [*sic*] Gravier 1906: 40–41.

####### Type locality.

“Grand Récif” (11.736°, 43.235°; estimated geolocation), Moucha Islands, Gulf of Tadjoura, Gulf of Aden, Indian Ocean.

####### Remarks.

As in the case of *Branchiomma claparedei* explained above, Gravier introduced the name *Branchiomma mushaensis* [sic] as new twice, first in 1906 (Gravier 1906: 40) and again in 1908 (Gravier 1908b: 94). This incurred some authors into error (e.g. Tovar-Hernández and Carrera-Parra 2011). The correct publication date is “1906” (see also Wehe and Fiege 2002).

###### Acromegalomma nechamae

Taxon classificationAnimaliaSabellidaSabellidae

(Knight-Jones, 1997) comb. n.

Megalomma nechamae Knight-Jones 1997: 319–320, fig. 4.

####### Type locality.

El Bilaiyim (= Ghor Blayim) lagoons, Sinai Peninsula, Gulf of Suez (28.55°, 33.24°; estimated geolocation).

###### Acromegalomma pacifici

Taxon classificationAnimaliaSabellidaSabellidae

(Grube & Örsted in Grube, 1859) comb. n.

Sabella pacifici Grube and Örsted in Grube 1859: 113.

####### Type locality.

Punta Arenas, Gulf of Nicoya, Costa Rica (9.976°, -84.852°; estimated geolocation).

####### Synonym.

*Pseudopotamilla panamica* Chamberlin 1919a: 268–269, plate 3 fig. 8 [subjective synonymy by Knight-Jones (1997)].

####### Remarks.

The authorship of the species is here considered as “Grube & Örsted *in* Grube, 1859”, according to Salazar-Vallejo and Eibye-Jacobsen (2012: 1398). The authorship appears clearly referred twice in the original publication as “*Gr. Örsd.*” (Grube 1859: 113, 120).

###### Acromegalomma perkinsi

Taxon classificationAnimaliaSabellidaSabellidae

(Tovar-Hernández & Salazar-Vallejo, 2006) comb. n.

Megalomma perkinsi Tovar-Hernández and Salazar-Vallejo 2006: 43–45, fig. 11.

####### Type locality.

Cape Lookout, North Carolina, Atlantic coast of the USA (34.62°, -76.54°; estimated geolocation).

###### Acromegalomma phyllisae

Taxon classificationAnimaliaSabellidaSabellidae

(Capa & Murray, 2009) comb. n.

Megalomma phyllisae Capa and Murray 2009: 205–208, figs 2A–E, 3, 4A–B, 5A.

####### Type locality.

Off Townsend Point, Corner Inlet, Victoria, Australia (-38.8°, 146.55°; original geolocation).

###### Acromegalomma pigmentum

Taxon classificationAnimaliaSabellidaSabellidae

(Reish, 1963) comb. n.

Megalomma pigmentum Reish 1963: 430–431, figs 15, 16A–I.

####### Type locality.

Bahía de San Quintín, Baja California, Mexico, Pacific Ocean (30.456°, -115.958°; estimated geolocation).

####### Synonym.

*Megalomma monoculata* [sic] Hartmann-Schröder 1965: 273–276, figs 276–278 [subjective synonymy by Knight-Jones (1997)].

###### Acromegalomma pseudogesae

Taxon classificationAnimaliaSabellidaSabellidae

(Mikac, Giangrande & Licciano, 2013) comb. n.

Megalomma pseudogesae Mikac et al. 2013: 1514–1515, fig. 3.

####### Type locality.

13 nautical miles off the coast of the Istrian Peninsula, Croatia, Gulf of Venice, Northern Adriatic Sea (45.2833°, 13.2667°; original geolocation).

###### Acromegalomma quadrioculatum

Taxon classificationAnimaliaSabellidaSabellidae

(Willey, 1905) comb. n.

Branchiomma quadrioculatum Willey 1905: 307, plate VII figs 168–169.

####### Type locality.

Aripu (= Arippu) Coral Reef, Sri Lanka, Gulf of Manaar, Indian Ocean (08.78°, 79.87°; estimated geolocation).

###### Acromegalomma roulei

Taxon classificationAnimaliaSabellidaSabellidae

(Gravier, 1908) comb. nov.

Branchiomma roulei Gravier 1907: 526 (*nomen nudum*); Gravier 1908a: 44.

####### Type locality.

Payta (= Paita), Peru, Pacific Ocean (-5.083°, -81.111°, estimated geolocation).

###### Acromegalomma splendidum

Taxon classificationAnimaliaSabellidaSabellidae

(Moore, 1905) comb. n.

Pseudopotamilla splendida Moore 1905: 564–566, plate XXXVII figs 23–27.

####### Type locality.

Kasaan Bay, center of Round Island, S. 10d W., 0.4 miles, Clarence Strait, Prince of Wales Island, Alexander Archipelago, SE Alaska, North Pacific Ocean (55.51°, -132.39°; estimated geolocation).

####### Synonyms.

*Pseudopotamilla anoculata* Moore 1905: 566–568, plate XXXVII figs 28–33 [subjective synonymy by Hartman (1959)]. *Branchiomma disparoculatum* Treadwell 1914: 223–224, plate 12 figs 44–46 [subjective synonymy by Hartman (1956)]. *Branchiomma burrardum* Berkeley 1930: 71, fig. 1 [subjective synonymy by Knight-Jones (1997)].

###### Acromegalomma suspiciens

Taxon classificationAnimaliaSabellidaSabellidae

(Ehlers, 1904) comb. n.

Branchiomma suspiciens Ehlers 1904: 62–63, plate IX figs 1–6.

####### Type locality.

French Pass, between D’Urville Island and north end of South Island, New Zealand (-40.922°, 173.837°; estimated geolocation).

###### Acromegalomma trioculatum

Taxon classificationAnimaliaSabellidaSabellidae

(Reish, 1968) comb. n.

Megalomma trioculatum Reish 1968: 226–228, fig. 5 (*1–10*).

####### Type locality.

Lagoon side of Engebi (= Enjebi) Island, Enewetak (= Eniwetok) Atoll, Ralik Chain, Marshall Islands, Pacific Ocean (11.658°, 162.235°; estimated geolocation).

###### Acromegalomma vesiculosum

Taxon classificationAnimaliaSabellidaSabellidae

(Montagu, 1813) comb. n.

Amphitrite vesiculosa Montagu 1813: 19–20, plate V fig. 1.

####### Type locality.

Original type locality at Kingsbridge Estuary, Devon, England (50.263°, -03.765°; estimated geolocation). Neotype designated by Knight-Jones (1997: 314) from St. Anthony, Cornwall, England (50.15°, -5.2667°; original geolocation misplaced, corrected here to 50.152°, -5.006°).

##### *Species inquirenda*

###### Megalomma vigilans

Taxon classificationAnimaliaSabellidaSabellidae

(Claparède, 1870) [unreplaced junior secondary homonym]

Branchiomma vigilans Claparède 1870: 501–503, plate XIV fig. 3.

####### Type locality.

Gulf of Naples, Mediterranean Sea (40.7°, 14.3°; estimated geolocation).

####### Remarks.

*Branchiomma vigilans* was described on the basis of three specimens from the Gulf of Naples, all of them found with their muddy tubes inserted among the dorsal chaetae of individuals of *Aphrodita aculeata* Linnaeus, 1758 (Claparède 1870). Afterwards the species was recorded on only a couple of occasions in the Western Mediterranean, first by Marion (1876), in the Gulf of Marseille and from 60–65 m (no habitat details), and later by Soulier (1903), who observed about ten specimens collected off Séte (Gulf of Aigues-Mortes) among the chaetae of *A. aculeata* specimens. Rioja (1923) attributed an empty sandy tube found among the dorsal chaetae of an *A. aculeata* collected in the region of Valencia to this species, but this record is very dubious, as not only was the worm not present but also the nature of the tube differed from that described by Claparède (1870). Moreover, no type material of *B. vigilans* is known to exist (Knight-Jones 1997, Giangrande and Licciano 2008, Tovar-Hernández and Carrera-Parra 2011) as Claparède normally did not deposit specimens in museums or collections (Fauchald 1989).

The species was transferred to *Megalomma* by Hartman (1959: 550), creating a junior secondary homonym of the tiger beetle *Megalomma vigilans* (Westwood, 1842) (see above), and has since remained a poorly known but valid taxon (Knight-Jones 1997, Tovar-Hernández and Salazar-Vallejo 2008). Giangrande and Licciano (2008) considered the species as being quite rare, probably due to its peculiar habitat, and in spite of stating that its real status needed confirmation, they also observed that it was likely a valid species. However, the species was subsequently omitted from the discussions on new Mediterranean species of *Megalomma* by Mikac et al. (2013) and Giangrande et al. (2015). The most recent reference to the species seems to be by Tovar-Hernández and Carrera-Parra (2011: 5), who wrote:

Megalomma vigilans *(Claparède, 1870) was originally found as an epibiont of the sea mouse* Aphrodita aculeata *Linnaeus, 1758, in the Mediterranean Sea, however, no new records of this association exist.* [...] *In the case of* M. vigilans, *the description is poor, the type is lost and there are no additional records*.

The described habitat of *Megalomma vigilans* is unusual, and there are no references of similar cases in the family Sabellidae. It is possible that the habitat is an artefact resulting from the collection process, and that the presence of the species on individuals of *Aphrodita aculeata* was the consequence of the rough treatment and mixing suffered by the biological material collected by grabs and trawls, or even during the processing of the samples. So, the presence of *M. vigilans* on *A. aculeata* could be a post-collection phenomenon, and not the natural habitat of the worm. It is difficult or even impossible to know if the records by Marion (1876) and Soulier (1903) refer to the same species as that collected and described by Claparède (1870) without studying the material, if still existing. There is a possibility that *M. vigilans* is not as uncommon as it seems, but that for some reason it has not been collected or recognised. For the time being, *M. vigilans* is here considered as a *species inquirenda*.

###### Megalomma pacificum

Taxon classificationAnimaliaSabellidaSabellidae

Johansson, 1927

Megalomma pacifica [*sic*] Johansson 1927: 130–131, textfig. 15.11.

####### Type locality.

Syntypes collected at Aranuka Island, outside the coral reef (0.14°, 173.56°; estimated geolocation), and Tapeteuea (= Tabiteuea) Island, inside the lagoon (-1.5°, 175.0°; estimated geolocation), both at Gilbert Islands, Kiribati, Pacific Ocean.

####### Remarks.

According to Fitzhugh (2002), *Megalomma pacificum* Johansson, 1927 probably belongs to the genus *Demonax* Kinberg, 1867 (a name recently replaced by *Parasabella* Bush, 1905 due to homonymy; see Tovar-Hernández and Harris 2010). The fact that the holotype has dried out (Knight-Jones 1997) and that the species has a remote type locality have likely prevented a formal redescription. The species was not included in the *Parasabella* species list given by Tovar-Hernández and Harris (2010), but its possible inclusion in *Parasabella* has been implicitly accepted by subsequent authors (Capa and Murray 2009, Tovar-Hernández and Carrera-Parra 2011).

## Supplementary Material

XML Treatment for Acromegalomma

XML Treatment for Acromegalomma acrophthalmos

XML Treatment for Acromegalomma adriaticum

XML Treatment for Acromegalomma bioculatum

XML Treatment for Acromegalomma carunculatum

XML Treatment for Acromegalomma cinctum

XML Treatment for Acromegalomma circumspectum

XML Treatment for Acromegalomma claparedei

XML Treatment for Acromegalomma coloratum

XML Treatment for Acromegalomma fauchaldi

XML Treatment for Acromegalomma georgiense

XML Treatment for Acromegalomma gesae

XML Treatment for Acromegalomma heterops

XML Treatment for Acromegalomma inflatum

XML Treatment for Acromegalomma interruptum

XML Treatment for Acromegalomma jubatum

XML Treatment for Acromegalomma kaikourense

XML Treatment for Acromegalomma lanigerum

XML Treatment for Acromegalomma lobiferum

XML Treatment for Acromegalomma longoventrale

XML Treatment for Acromegalomma messapicum

XML Treatment for Acromegalomma miyukiae

XML Treatment for Acromegalomma modestum

XML Treatment for Acromegalomma multioculatum

XML Treatment for Acromegalomma mushaense

XML Treatment for Acromegalomma nechamae

XML Treatment for Acromegalomma pacifici

XML Treatment for Acromegalomma perkinsi

XML Treatment for Acromegalomma phyllisae

XML Treatment for Acromegalomma pigmentum

XML Treatment for Acromegalomma pseudogesae

XML Treatment for Acromegalomma quadrioculatum

XML Treatment for Acromegalomma roulei

XML Treatment for Acromegalomma splendidum

XML Treatment for Acromegalomma suspiciens

XML Treatment for Acromegalomma trioculatum

XML Treatment for Acromegalomma vesiculosum

XML Treatment for Megalomma vigilans

XML Treatment for Megalomma pacificum
